# Comparative Efficacy of Interventions for the Management of Oral Submucous Fibrosis: A Systematic Review and Network Meta-Analysis

**DOI:** 10.3390/jpm12081272

**Published:** 2022-08-01

**Authors:** Divya Gopinath, Lai Mong Hui, Sajesh K. Veettil, Athira Balakrishnan Nair, Mari Kannan Maharajan

**Affiliations:** 1 Clinical Oral Health Sciences, International Medical University, Kuala Lumpur 57000, Malaysia; 2 School of Postgraduate Studies, International Medical University, Kuala Lumpur 57000, Malaysia; lai.monghui@student.imu.edu.my; 3 Department of Pharmacotherapy, College of Pharmacy, University of Utah, Salt Lake City, UT 84112, USA; sajesh.veettil@pharm.utah.edu; 4 Independent Researcher, Manipal 576104, India; athira.b@manipal.edu; 5 School of Pharmacy, University of Nottingham Malaysia, Semenyih 43500, Malaysia; marikannan.maharajan@nottingham.edu.my

**Keywords:** oral submucous fibrosis, management, treatment, medical interventions, agents, systematic review, network meta-analysis

## Abstract

**Introduction:** Oral submucous fibrosis (OSMF) is a chronic premalignant condition and is characterized by fibroblastic change of lamina propria and stiffness of oral mucosa. Though there are several treatment options available, the best agent is not yet identified. This study assessed the comparative efficacy and safety of medical interventions in the management of OSMF. **Methods:** A systematic review was performed to identify randomized controlled trials (RCTs) that compared the efficacy of interventions for OSMF with each other, or placebo. A network meta-analysis was performed, and the interventions were ranked according to their efficacy based on the surface under the cumulative ranking. (PROSPERO Registration no: CRD42021255094). **Results:** Thirty-two RCTs comprising 2063 patients were eligible for quantitative analysis. In terms of therapeutic efficacy in the improvement of mouth opening Oxitard, a herbal formulation was ranked as the most efficacious agent, [MD, 10.29 (95%CI 6.34–14.25)] followed by combination therapy of Lycopene with corticosteroids and hyaluronidase [MD, 7.07 (95%CI 1.82–12.31)]. For improvement of burning sensation aloe vera was ranked first [MD, 6.14 (95%CI 4.58–7.70)] followed by corticosteroids with antioxidants [MD, 6.13 (95%CI 4.12–8.14)] and corticosteroids in combination with hyaluronidase with antioxidants [MD, 5.95 (95%CI 3.79–8.11)]. In terms of safety, most of the drugs were reported to cause mild adverse effects only. Significant inconsistencies could be identified in the analysis for both the outcomes assessed and were further explored. **Conclusions:** Our study highlighted the potential efficacy of several agents over placebo in the improvement of mouth opening and burning sensation in OSMF patients. However, the RCTs lacked methodological soundness. Well-designed studies with a larger number of participants with a rigorous randomization process and stringent methodology are recommended to strengthen the results obtained, which may help to construct a clinical guideline for OSMF management.

## 1. Introduction

Oral submucous fibrosis (OSMF) is a chronic premalignant condition and is characterized by fibroblastic change of lamina propria and stiffness of oral mucosa [1] eventually leading to mucosal stiffness and functional morbidity of the oral cavity. The malignant transformation potential of OSMF ranges between 8 and 10% [2]. The signs and symptoms include a burning sensation and the inability of opening the mouth leading to issues in speech and swallowing [3]. Histopathologically, OSMF is characterized by intense fibrosis, which affects the oral cavity, pharynx, and upper third of the esophagus [2,3].

Several pharmacotherapeutic options have been tried and tested to relieve the signs and symptoms of OSMF. The pharmacological agents include corticosteroids, enzymes, interferon-gamma, antioxidants, methylxanthine derivatives, placental extracts, immune milk, turmeric, colchicine, tea pigments, aloe vera, and spirulina. Corticosteroids including hydrocortisone [4], triamcinolone [5,6,7] and dexamethasone [8] function as a suppressor of inflammatory response and minimizes fibrosis. Enzymes including hyaluronidase [7,8], chymotrypsin [9] and collagenase [10] are used solely or in combination with other agents to amplify the effectiveness of the regimen. Placental extracts in the management of OSMF help by biogenic stimulation through metabolic regenerative process [11,12]. The role of interferon-gamma involves altering collagen formation and it has been shown to be superior to other agents in its ability to reverse the symptoms [13]. Antioxidants are believed to improve the symptoms by protecting cells from damage from free radicals. Agents such as lycopene [14,15], spirulina [16], turmeric [17], aloe vera [18] and green tea [19] possess antioxidant properties which are believed to aid in the relief of symptoms. Other agents such as pentoxifylline [20,21], immune milk [22], and colchicine [23] are also observed to help in the relief of OSMF symptoms. Besides medical intervention, physiotherapy, as well as surgical methods, are observed to benefit the management of OSMF [24]. 

The comparative efficacy of these available interventions has not been assessed yet and the best agent is yet to be identified. The last Cochrane review which was performed more than a decade ago in 2008 concluded that there is no reliable evidence for the effectiveness of any specific interventions for the management of oral submucous fibrosis [25].

Considering the recent advancements in pharmacotherapeutics in the management of OSMF, there are still no definitive recommended treatment guidelines for OSMF management. Decision-making in the management of oral disease especially when it carries an increased risk of malignant transformation should be based on high-quality evidence. Though meta-analysis (MA) helps to synthesize the results of various trials into a single meaningful outcome, thereby replacing the requirement of a large or complete trial [26], conventional MA is restricted to synthesizing trials with no more than two interventions for a single disease. Overcoming this limitation, network meta-analysis (NMA) allows the synthesis of trials with different treatment options to be compared through the evaluation of direct or indirect evidence. NMA enables the establishment of the most effective intervention for a particular problem that has multiple treatment options [27,28]. The purpose of this SR and NMA of RCTs was to identify the most effective medical intervention in reducing the clinical symptoms of OSMF. This will aid physicians and other healthcare practitioners to determine which intervention or which combination is best to be used in the management of OSMF in terms of effectiveness and safety.

## 2. Materials and Methods

This systematic review consisted of a compilation of randomized controlled trials on medical interventions for OSMF treatment and was performed following the general principles outlined in the Cochrane Handbook for Systematic Reviews of Interventions [29]. The systematic review is reported in accordance with the Preferred Reporting Items for Systematic Reviews and Meta-Analyses (PRISMA) extension statement for network meta-analysis [30]. This protocol was registered in the PROSPERO international prospective register of the systematic reviews (Prospero id: CRD42021255094).

### 2.1. Data Source and Search Strategy

We systematically searched for relevant studies in databases including Ovid-Medline, Pubmed and Cochrane Central Register of Controlled Trials. Besides that, we also performed a manual search for additional studies in published reviews. The search was performed by using subject headings, free-text terms for oral submucous fibrosis and relevant interventions to identify relevant randomized controlled trials. The search strategy was developed for Medline and was modified for other databases as shown in Supplementary Table S1. The detailed search algorithm is provided in Supplementary Table S2.

### 2.2. Subject Selection Criteria

Studies included were randomized controlled trials that met the following eligibility criteria based on the Participants, Intervention, Comparator and Outcomes (PICO) as follows:

Participants (P): Adult patients diagnosed with OSMF
Intervention (I): Any class of medicinal intervention used in the management of OSMF
Comparator (C): Any other medicinal intervention or placebo
Outcomes (O): Primary Outcome: Mouth opening; Secondary Outcome: Burning sensation, cheek flexibility, tongue protrusion and other outcomes or side effects and
Study design (S): Randomized controlled trials (RCT)

### 2.3. Data Extraction and Quality Assessment

Titles and abstracts were screened and reviewed independently by (LMH & DVG), followed by full-text reading. Ineligible studies were excluded after full-text reading. The data extraction form was created in accordance with the guidelines in the Cochrane Handbook for Systematic Reviews of Interventions by the consensus of both reviewers. Data extraction was conducted independently in duplicate by two reviewers and if multiple publications of the same trial were found, only the most recent and relevant data from these publications were included. We created identical data extraction forms for each class of drugs or treatments. The data extracted from studies were then separated into the following sections: study characteristics, population characteristics, intervention characteristics and outcome definitions and measures. For all outcomes, we followed the intention-to-treat principle [30] where we took the initial number of participants randomized to each trial arm and performed the analyses irrespective of how the data analysis was conducted by the authors of the original trials.

After data extraction was completed, we assessed the risk of bias within each study. The assessment was carried out independently by the same two reviewers using the revised Cochrane risk of bias tool (RoB 2.0) [31] and we resolved disagreements regarding the risk of bias through discussions.

### 2.4. Strategy for Data Synthesis

For the primary analysis, data were analyzed in accordance with the intention-to-treat principle. We used 95% confidence intervals and mean differences as a summary statistic. For direct comparison, a standard, pairwise meta-analysis was conducted with a random-effects (DerSimonian and Laird) model [30,32]. For direct comparison which involved two or more studies, we used I^2^ statistics to determine the heterogeneity between trials where an I^2^ estimate of more than 50% indicated a substantial level of heterogeneity [30,32]. Direct and indirect evidence were then combined and synthesized using random-effects network meta-analysis using consistency or inconsistency models [33,34] and the inconsistencies between the direct and indirect estimates which is known as network inconsistency assumption was assessed using a global inconsistency test by fitting design-by-treatment in the inconsistency model [33]. A comparison-adjusted funnel plot was used to assess publication bias. Statistical analysis and graph plotting were performed using Stata version 15.0 (StataCorp, College Station, TX, USA).

## 3. Results

### 3.1. Literature Search and Study Selection

**Figure 1:**
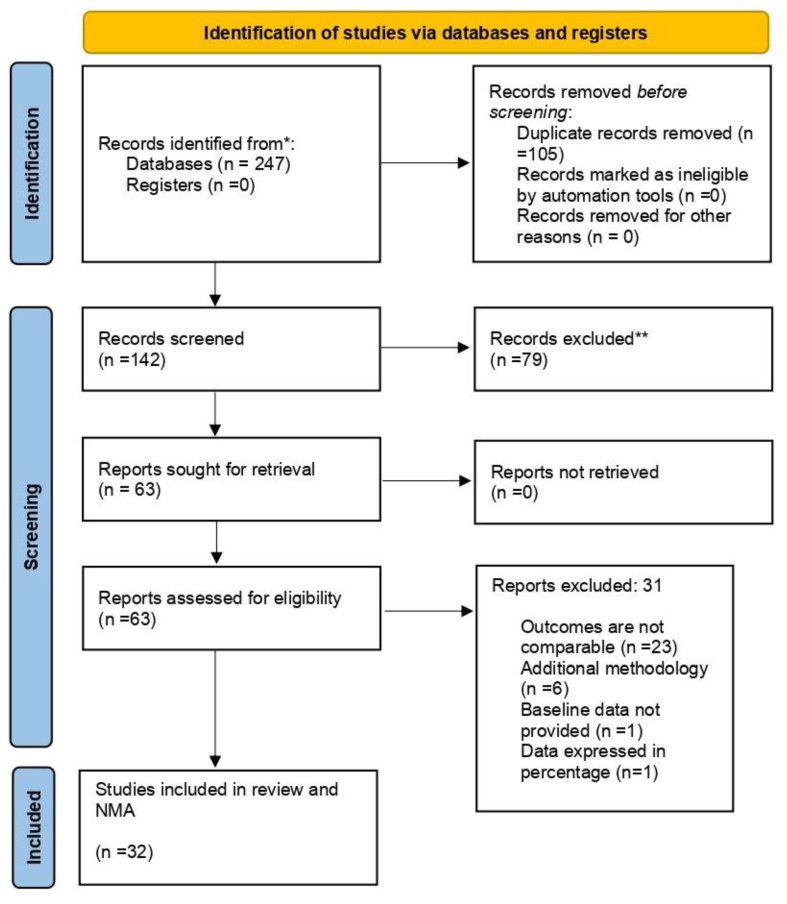
Prisma Flow Chart.

Figure 1 shows the process of literature search in accordance with the PRISMA guidelines. The database search yielded a total of 247 studies. After the removal of duplicated studies, 142 studies were selected for the title and abstract screening and after the screening, 79 studies were excluded as they did not fulfil the inclusion criteria. The remaining 63 studies were selected for full-text screening and out of the 63 studies, 32 studies were selected as they met the eligibility criteria and were included in the NMA. The list of studies excluded with reasons can be referred to in the Supplementary Table S3. The detailed flow chart is shown as Figure 1.

### 3.2. General Characteristics of the Included Studies

The general characteristics of the included studies are listed in Supplementary Table S4 [6,14,18,35,36,37,38,39,40,41,42,43,44,45,46,47,48,49,50,51,52,53,54,55,56,57,58,59,60,61,62,63]. A total of 2063 patients are involved in the trials with most of the trials conducted in India, 1 trial conducted in Pakistan [50] and 2 trials conducted in China [6,47]. The age of participants ranged from 15–70 years with most of them being male. The reported duration of treatment in the included studies ranged from 5 weeks–to 6 months with a follow-up period of 3 weeks–9 months.

### 3.3. Clinical Parameters

The clinical parameters assessed mentioned in the included randomized controlled trials consist of mouth opening, burning sensation, ulceration reduction, relief of fibrous bands, tongue protrusion, cheek flexibility, pain, speech, and swallowing difficulty. Out of 32 studies that measured mouth opening, 18 studies used vernier calipers [18,36,38,39,40,41,42,43,46,50,51,52,53,55,58,59,61,63], 3 studies used geometric scale/divider [14,39,60], 2 studies used sliding calipers [6,47], 3 studies used a metal scale/divider [37,56,62] and 7 studies did not mention any particular scale or divider [35,44,45,48,49,54,57]. Out of the 32 studies, 15 studies evaluated the burning sensation. Most of the studies reported evaluation of burning sensation using the visual analogue scale (VAS) [6,14,18,36,37,38,39,40,41,42,46,47,48,49,51,52,54,55,58,59,60,61,62,63] while some studies evaluated burning sensation categorizing as absent, present and reduced [43,44,45,56,57] and 1 study evaluated the sensation as mild, moderate and severe [53]. Some studies reported pain, difficulty in speech and swallowing categorizing as absence, presence and reduction [44,45,48,57].

### 3.4. Comparative Efficacy of Interventions in the Reduction of Mouth Opening (Primary Outcome)

The primary outcome identified was the improvement in mouth opening (difference between post-treatment and pre-treatment). Most of the included studies demonstrated statistically significant improvement in mouth opening [14,35,36,38,40,41,42,44,45,46,47,48,49,50,51,52,53,55,56,57,58,59,60,61,62].

**Figure 2:**
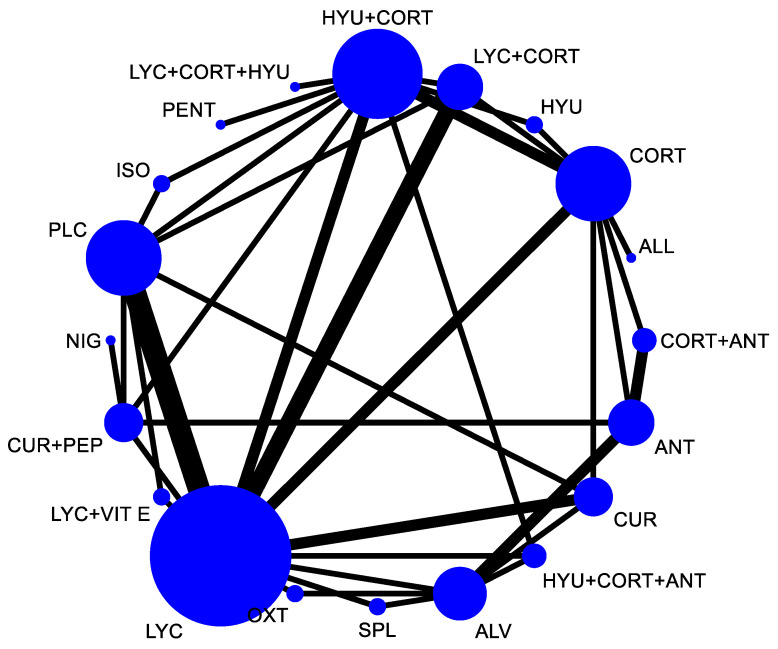
Network plot comparing the comparative efficacy of interventions used in the management of OSMF (mouth opening). Abbreviations: HYU—hyaluronidase; CORT—corticosteroid; LYC—lycopene; ALL—allicin; ANT—antioxidant; CUR—curcumin; ALV—aloe vera; SPL—Spirullina; OXT—Oxitard; VIT E—vitamin E; PEP—piperine; NIG—Nigella sativa; PLC—placebo; ISO—isoxsuprine; PENT—pentoxifylline.

In the network plot as shown in Figure 2, the size of the node indicates the total participants in that intervention and the thickness of the lines indicates the number of trials included. For the assessment of comparative efficacy, out of the 19 interventions included in the analysis for mouth opening, eleven interventions were found significant in the improvement of mouth opening as compared to placebo.

**Figure 3:**
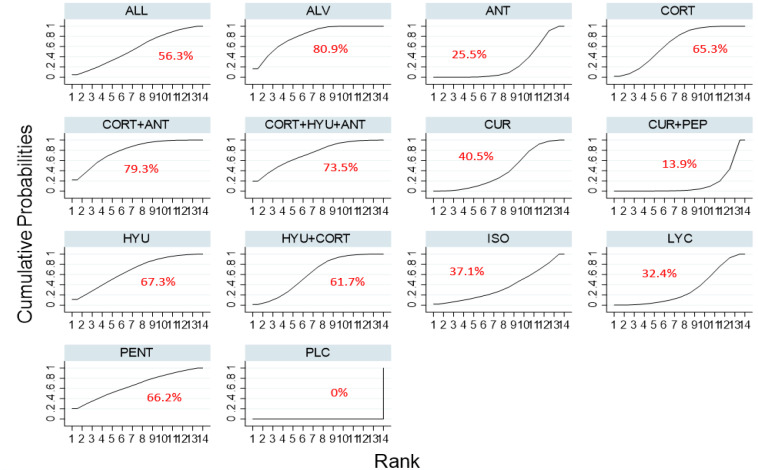
SUCRA ranking curve for the comparative efficacy of different interventions used in the management of OSMF (mouth opening). Abbreviations: HYU—hyaluronidase; CORT—corticosteroid; LYC— lycopene; ALL—allicin; ANT—antioxidant; CUR—curcumin; ALV—aloe vera; PEP—piperine; PLC—placebo; ISO—isoxsuprine; PENT—pentoxifylline.

**Figure 4:**
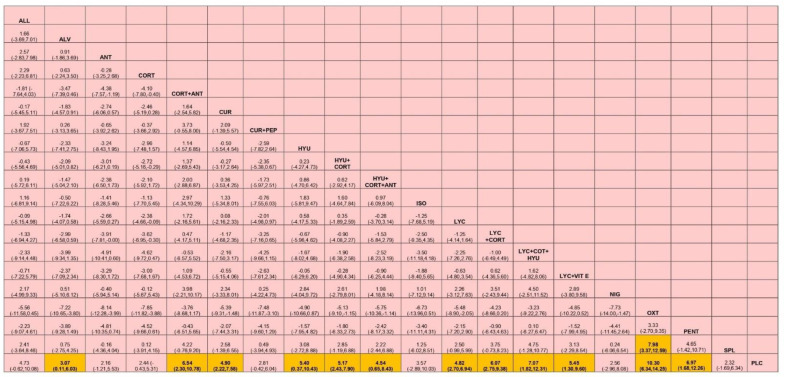
League table showing the results of the network meta-analyses comparing the effects of all drugs in improving mouth opening (MD) and 95% credible intervals. Abbreviations: HYU—hyaluronidase; CORT—corticosteroid; LYC—lycopene; ALL—allicin; ANT—antioxidant; CUR—curcumin; ALV—aloe vera SPL—Spirulina; OXT—Oxitard; VIT E—vitamin E; PEP—piperine; NIG—Nigella sativa; PLC—placebo; ISO—isoxsuprine; PENT—pentoxifylline.

Based on the SUCRA ranking of efficacy, Oxitard was shown to be the most effective among all the interventions in improving mouth opening [MD, 10.29 (95%CI 6.34–14.25)] followed by the combination therapy of lycopene, hyaluronidase, and corticosteroids [MD, 7.07 (95%CI 1.82–12.31)]. The mean differences between pre and post treatment of each of the interventions tested are shown in Table 1. The lowest ranked intervention was the treatment by antioxidants [MD, 2.16 (95%CI-1.21–5.53)] whereas corticosteroids were slightly better than antioxidants therapy [MD, 2.44 (95%CI.43–5.31)]. The SUCRA ranking graph is shown in Figure 3 and the league table, Figure 4.

### 3.5. Secondary Outcomes

#### 3.5.1. Comparative Efficacy of Interventions in the Reduction of Burning Sensation

**Figure 5:**
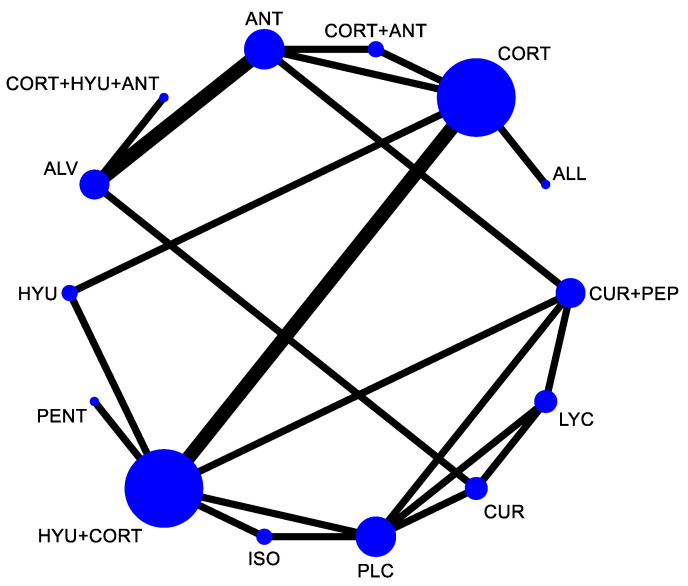
Network plot comparing the comparative efficacy of interventions used in the management of OSMF (burning sensation). Abbreviations: HYU—hyaluronidase; CORT—corticosteroid; LYC—lycopene; ALL—allicin; ANT—antioxidant; CUR—curcumin; ALV—aloe vera; PEP—piperine; PLC—placebo; ISO—isoxsuprine; PENT—pentoxifylline.

**Figure 6:**
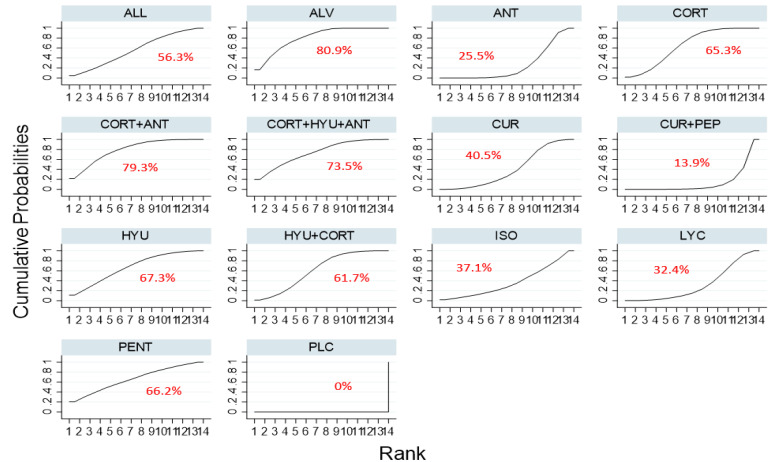
SUCRA ranking curve for the comparative efficacy of different interventions used in the management of OSMF (Burning sensation) Abbreviations: HYU—hyaluronidase; CORT—corticosteroid; LYC— lycopene; ALL—allicin; ANT—antioxidant; CUR—curcumin; ALV—aloe vera; PEP—piperine; PLC—placebo; ISO—isoxsuprine; PENT—pentoxifylline.

**Figure 7:**
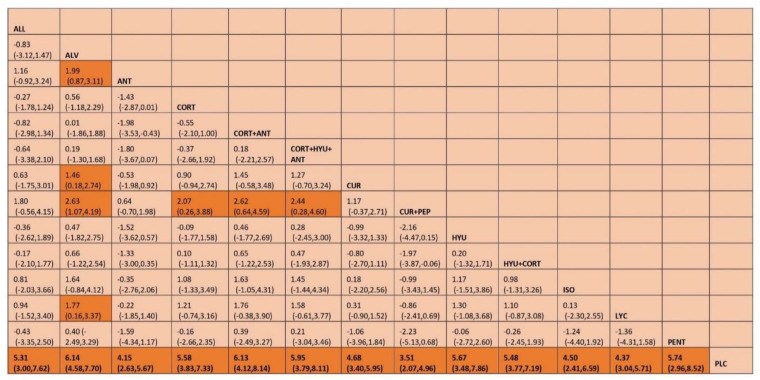
League table showing the results of the network meta-analyses comparing the effects of all drugs in improving mouth opening (MD) and 95% credible intervals Abbreviations: HYU—hyaluronidase; CORT—corticosteroid; LYC—lycopene; ALL—allicin; ANT—antioxidant; CUR—curcumin; ALV—aloe vera; PEP—piperine; PLC—placebo; ISO—isoxsuprine; PENT—pentoxifylline.

Out of the 32 included studies, 29 studies evaluated the burning sensation. 13 interventions were connected in the network plot as shown in Figure 5 and eleven interventions were found to be effective in relieving the mouth burning sensation compared to placebo. Among these interventions, aloe vera was shown to be the most effective in burning sensation reduction [MD, 6.14 (95%CI 4.58–7.70)] followed by combination therapy of corticosteroid and antioxidants [MD, 6.13 (95%CI 4.12–8.14)]. The lowest ranked intervention was the combination treatment of curcumin and piperine [MD, 3.51 (95%CI 2.07–4.96)] whereas treatment by antioxidants a showed a slightly better treatment effect [MD, 4.15 (95%CI 2.63–5.67)]. The SUCRA ranking table for burning sensation is shown in Table 2 and the ranking graph is provided in Figure 6 and league table as Figure 7.

We used the “global wald test” as one of the methods to determine the inconsistencies for the whole network. The differences in design used in each study were considered as interaction terms (called design by treatment interaction inconsistency model) for the inconsistency model to estimate the treatment effect of each intervention. The test implied that the network model for both mouth opening and burning sensations are inconsistent (Supplementary Tables S5 and S6)

We used the node splitting method to explore the inconsistency (Supplementary Table S7). However, we were not able to exclude the inconsistency in this model. Hence, we present the results based on the principles of this inconsistency model described by the Cochrane workshop [64]. The direct evidence was first separated from the network of indirect evidence and then the evidence is compared with each other one at a time to determine the inconsistencies [65,66]. The discrepancies between the estimates of relative treatment effects from these two sets of trials (direct and indirect) indicate the level of inconsistency [67]. We also performed the loop-specific approach to assess the inconsistency in a network. This approach corresponds to differences between direct and various indirect effect estimates between the same intervention comparison [68]. We found 21 closed triangular loops (formed by three treatments) and 4 quadrilateral groups in the primary outcome network (Supplementary Figure S1).

#### 3.5.2. Tongue Protrusion

Among the 32 included studies, 12 studies reported tongue protrusion. Most of the studies showed statistically significant results [37,45,46,48,57,58,62] and for those studies with non-significant results, the results were still positive outcomes, although they were not enough to be considered statistically significant. For example, according to a Sudarshan R et al. [18] and Singh N et al. [52], the improvements in tongue protrusion are non-significant, but the aloe vera group showed a better improvement as compared to the antioxidants group. According to a study conducted by Piyush P et al., the combination of curcumin and piperidine showed improvement in tongue protrusion followed by lycopene and placebo capsules although the improvement is not statistically significant [60].

#### 3.5.3. Cheek Flexibility

Among the 32 included studies, only 6 studies reported cheek flexibility. All the studies demonstrated positive outcomes on cheek flexibility and some studies demonstrated statistically significant results [37,58,60] while others studies showed results that are not enough to cause a significant difference. For example, according to a study by Sudarshan R et al. [18] and Singh N et al. [52], aloe vera showed a better outcome in cheek flexibility than antioxidants although the results were not statistically significant.

#### 3.5.4. Other Outcomes

Besides the outcomes mentioned, few studies reported outcomes such as pain while opening the mouth, difficulty in speech as well as swallowing, as well ulcerations and blanching. The studies demonstrated improvement in all these outcomes [14,35,38,41,43,44,45,48,49,53,57,58].

### 3.6. Side Effects

Overall, very few studies have reported the side effects of the interventions tested. One study reported the development of severe burning sensation and oral ulcers 1 week after initiation of lycopene therapy [14]. Another study reported nausea from aloe vera gel therapy and an increase in appetite following antioxidant therapy [18]. Two studies reported abdominal discomfort from Oxitard [45,57]. Gastrointestinal side effects such as dyspepsia, nausea, vomiting, and bloating as well as central nervous system side effects such as headache, anxiety, tremor, and confusion from pentoxifylline were reported in one study [55]. One study reported hypertrichosis from triamcinolone injection [6]. Curcumin lozenges caused yellowish coating over the teeth and dorsum of the tongue which was managed through oral prophylaxis procedures and maintenance of oral hygiene [51]. One study reported an increase in appetite after the usage of aloe vera gel [37]. Mild gastric irritation from curcumin in the form of bloating and flatulence was reported in another study [60]. The combination therapy of turmeric tablets and turmeric mouthwash has been reported to cause facial swelling and erythema on the palms 1 day after therapy initiation and abdominal discomfort, but these patients were lost from follow-up later in the study [62].

### 3.7. Quality of Included Studies

**Figure 8:**
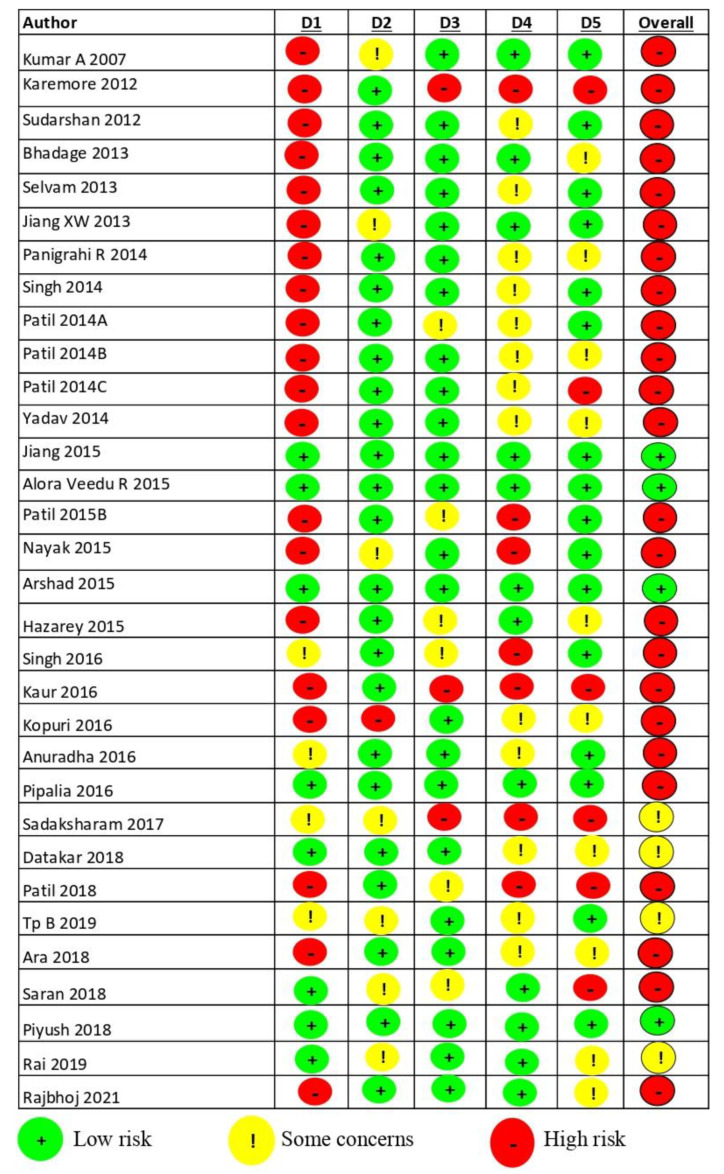
Summary of risk of bias.

The studies were evaluated on their quality using the Cochrane risk of bias tool for the primary outcome (Figure 8). Only 4 studies [35,47,50,60] among the 32 included studies demonstrated a low risk of bias. A total of 24 studies [6,14,18,36,37,38,39,40,41,42,43,44,45,46,48,49,51,52,53,54,57,58,59,63] showed a high risk of bias while 4 studies [55,56,61,62] showed some concerns of bias with major bias in the randomization process followed by outcome measurement and reporting bias.

### 3.8. Pairwise Meta-Analysis

#### 3.8.1. Mouth Opening

We also performed individual pairwise meta-analysis for lycopene, combination therapy of corticosteroids and hyaluronidase, curcumin, and aloe vera as these were evaluated in several trials. The results showed that lycopene demonstrated the best results in terms of improving mouth opening compared with other interventions although there was significant heterogeneity in the results obtained (MD, 1.38; 95% CI, 0.44–2.33; I^2^ > 95%). However, for the pairwise comparison of other agents, the results were insignificant in terms of mouth opening improvement (Supplementary Figures S2–S5).

#### 3.8.2. Burning Sensation

We conducted pairwise meta-analysis for lycopene and curcumin individually and the results showed that the pooled pairwise comparison of these agents with control groups (other interventions) was insignificant in terms of reduction of burning sensation, (Supplementary Figures S6 and S7).

Forest plots for all meta-analysis comparisons indicated publication bias (Supplementary Figures S8–S11).

## 4. Discussion

Even though a wide variety of interventions are available, currently there is no consensus or recommended guidelines for the management of OSMF [69,70,71]. To the best of our knowledge, this is the first network meta-analysis reported on the comparative efficacy of available interventions in the management of OSMF. The network meta-analysis revealed that most of the interventions studied are superior to placebo in improving the clinical symptoms including the reduction in mouth opening and burning sensation. None of these interventions was superior to each other except for Oxitard in improving the mouth opening. However, aloe vera was shown to be superior to curcumin in relieving the burning sensation.

Based on SUCRA ranking, Oxitard was shown to be most effective in improving mouth opening compared to placebo. The Oxitard is an ayurvedic medicine in the form of capsules containing the herbal extracts of Mangifera indica, Withania somnifera, Daucus carota, Glycyrrhiza glabra, Vitis vinifera, powders of Emblica officinalis and Yashada bhasma, and oils of Triticum sativum. These components have been shown to possess antibacterial, antiviral, and inflammation suppressant properties that can help to reduce burning sensation and wound healing. Moreover, the compound also contains minerals that help to reduce oxidative stress [72]. However, only three trials have tested this intervention until now and side effects have been reported.

Aloe vera was shown to be most effective in burning sensation relief whereas it was not as efficacious as many other agents in improving mouth opening. Aloe vera contains vitamins, enzymes, minerals, sugars, lignin, saponins, salicylic acids, and amino acids. Vitamin A, C, and E are antioxidants that can aid in scavenging free radicals [73,74] and enzymes such as bradykinase which help in reducing inflammation through topical application. It also contains polysaccharides with anticancer, anti-inflammatory, and wound-healing properties [75]. These properties might explain the reason for its efficacy in relieving the burning sensation.

Several studies have utilized lycopene as a single agent as well as in combination with hyaluronidase along with various corticosteroids. The mechanism of action of lycopene involves its antioxidant effect by quenching free radicals physically and chemically, thus contributing to the protection of cellular components from damage by highly reactive oxygen species (ROS) [76,77]. A study on the combination of corticosteroid-dexamethasone and hyaluronidase with lycopene demonstrated that the combination therapy was superior to lycopene in relieving OSMF symptoms among 60 OSMF patients [61]. In another study on lycopene, combination therapy of lycopene and hyaluronidase and placebo among 45 OSMF patients, lycopene and combination therapy of lycopene and hyaluronidase was found to show statistically significant improvement in mouth opening and burning sensation as compared to placebo. However, when those agents were compared with each other (lycopene and lycopene-hyaluronidase), lycopene as a single agent was observed to result in a better outcome as compared to the combination therapy [78]. Our NMA revealed that a combination of lycopene, hyaluronidase, and corticosteroids, the combination of lycopene and corticosteroids, and a combination of lycopene and vitamin E were quite effective in improving mouth opening. However, lycopene as a single agent was ranked inferior to the combination therapies in the improvement of mouth opening. For relief of burning sensation, lycopene was ranked inferior to many other therapies. Although it is not the most efficacious intervention in the improvement of mouth opening and burning sensation, it might be considered an agent to be used in OSMF management since it is more tolerable as patients on lycopene did not report any side effects.

The combination therapy of corticosteroids with hyaluronidase was also widely studied. Hyaluronidase is an enzyme that can degrade hyaluronic acid which is an important component in the extracellular matrix [79]. Therefore, this ability allows it to degrade collagen and decrease the formation of collagen [46]. In addition, through degradation of hyaluronic acid, it reduces the intracellular viscosity and stimulates the breakdown of the fibrinous coagulum [35,80]. The combination therapy of corticosteroids and hyaluronidase tends to allow better penetration of corticosteroids thus leading to better results. However, there are drawbacks to the therapy such as pain while injecting, the formation of scars due to needle prick and a higher chance of relapse. Corticosteroids in general function by suppressing inflammatory reactions and dissipating the by-products of inflammation. This, in turn, reduces fibroblastic proliferation and collagen deposition by enhancing phagocytic activity and thereby preventing fibrosis of mucosa [35,80]. Our NMA also demonstrated that corticosteroids showed better results when used in combination therapy with lycopene, hyaluronidase, or antioxidants when compared to their use as a solo therapy. For burning sensation relief, corticosteroids in combination with antioxidants or hyaluronidase showed better results than any of these used alone. Therefore, it can be proposed that combination therapy of corticosteroids with other agents can be used in OSMF management since the overall result is quite effective. However, newer, and advanced methods have to be explored for intralesional delivery due to the tendency for fibrosis [81].

Another agent, which has been used as a single agent as well as a combination therapy with piperine is curcumin which has demonstrated its effectiveness in relieving OSMF signs and symptoms, although the number of studies is still quite limited. The effectiveness of curcumin might be contributed by its antioxidant and anti-inflammatory properties that highlight its use in the treatment and prevention of inflammation and oxidative stress [82].

Besides the agents mentioned, there are other agents studied in the trials selected including pentoxifylline, spirulina, isoxsuprine, as well as supplements such as vitamin E, allicin, and antioxidants which have been proven efficacious in the secondary outcomes we assessed. However very few studies had reported these outcomes and because of the heterogeneity, these secondary outcomes could not be quantitatively analyzed.

Although there were some adverse effects reported, most of the interventions investigated for the management of OSMF were well tolerated. Intralesional corticosteroids were generally well tolerated although there was one study where a patient withdrew from the study due to hypertrichosis from triamcinolone injection. Aloe vera gel was reported to cause increased appetite as well as nausea. Although Oxitard has shown high efficacy in OSMF treatment, it has been reported to cause abdominal discomfort. Curcumin despite being a traditional agent, cause gastric irritation, allergic reaction as well as discoloration to the oral cavity. Pentoxifylline has been shown to contribute to gastrointestinal and central nervous system problems. There were no reported side effects from lycopene despite being the most studied agent. The chronic course of the OSMF treatment which requires long-term use of various medications might increase the risk of side effects and can affect the compliance of patients to the treatment. Hence, lycopene which has the lowest tendency to cause side effects can be considered the best agent in terms of safety.

Our study has some limitations. We have identified inconsistency in the network model and hence have analyzed and presented the results according to the inconsistency model of NMA; hence the results of this NMA must be interpreted with caution. Moreover, most of the trials presented with high risk of bias which was attributed mostly to the improper randomization process and the improper outcome measurement. Another drawback was the limited sample sizes of the included studies which can lead to the inaccurate representation of the results. Going forward, given that most of the comparisons in the NMA were downgraded due to study limitations (risk of bias) and imprecision, well-designed randomized controlled trials with adequate sample sizes are highly recommended.

## 5. Conclusions

This network meta-analysis highlights that several commonly used interventions are effective in improving the mouth opening in OSMF patients compared to placebo, although they are not superior to each other. Aloe vera can be considered the most effective agent in reducing the burning sensation. Although this network meta-analysis offers certain evidence to aid the selection of interventions in patients with OSMF, further studies are recommended with a focus on strengthening this evidence with more stringent methodology and larger sample size, which may help to formulate clinical guidelines for the management of this potentially malignant condition.

## Figures and Tables

**Table 1:** SUCRA ranking table for the comparative efficacy of different interventions used in the management of OSMF (mouth opening).

Intervention	All RCTs		
	Mean Difference [95% CI]	*p*-Value	SUCRA Rank
OXT	10.30 [6.34–14.25]	0.000	1
LYC+CORT+HYU	7.07 [1.82–12.31]	0.008	2
PENT	6.97 [1.68–12.26]	0.010	3
CORT+ANT	6.54 [2.30–10.78]	0.002	4
LYC+CORT	6.07 [2.75–9.38]	0.000	5
LYC+VIT	5.45 [1.30–9.60]	0.010	6
HYU	5.40 [0.37–10.43]	0.035	7
HYU+CORT	5.17 [2.43–7.90]	0.000	8
CUR	4.90 [2.22–7.58]	0.000	9
LYC	4.82 [2.70–6.94]	0.000	10
ALL	4.73 [−0.62–10.08]	0.083	11
HYU+CORT+ANT	4.54 [0.65–8.43]	0.022	12
ISO	3.57 [−2.89–10.03]	0.279	13
ALV	3.07 [0.11–6.03]	0.042	14
NIG	2.56 [−2.96–8.08]	0.363	15
CUR+PER	2.81 [−0.42–6.04]	0.088	16
SPL	2.32 [−1.69–6.34	0.257	17
CORT	2.44 [−0.43–5.31]	0.095	18
ANT	2.16 [−1.21–5.53]	0.210	19

Abbreviations: HYU—hyaluronidase; CORT—corticosteroid; LYC— lycopene; ALL—allicin; ANT—antioxidant; CUR—curcumin; ALV—aloe vera; SPL—Spirullina; OXT—Oxitard; VIT E—vitamin E; PEP—piperine; NIG—Nigella sativa; PLC—placebo; ISO—isoxsuprine; PENT—pentoxifylline.

**Table 2:** SUCRA ranking table for the comparative efficacy of different interventions used in the management of OSMF (burning sensation).

Intervention	All RCTs		
	MD [95% CI]	*p*-Value	SUCRA Rank
ALV	6.14 [4.58–7.70]	0.00	1
CORT+ANT	6.13 [4.12–8.14]	0.00	2
CORT+HYU+ANT	5.95 [3.79–8.11]	0.00	3
HYU	5.67 [3.48–7.86]	0.00	4
PENT	5.74 [2.96–8.52]	0.00	5
CORT	5.58 [3.83–7.33]	0.00	6
HYU+CORT	5.48 [3.77–7.19]	0.00	7
ALL	5.31 [3.00–7.62]	0.00	8
CUR	4.68 [3.40–5.95]	0.00	9
ISO	4.50 [2.41–6.59]	0.00	10
LYC	4.37 [3.04–5.71]	0.00	11
ANT	4.15 [2.63–5.67]	0.00	12
CUR+PEP	3.51 [2.07–4.96]	0.00	13
PLC	0	0.00	14

Abbreviations: HYU—hyaluronidase; CORT—corticosteroid; LYC—lycopene; ALL—allicin; ANT—antioxidant; CUR—curcumin; ALV—aloe vera; PEP—piperine; PLC—placebo; ISO—isoxsuprine; PENT—pentoxifylline.

## Data Availability

Not applicable.

## Supplementary Materials

The following supporting information can be downloaded at: https://www.mdpi.com/article/10.3390/jpm12081272/s1, Table S1 Search strategy, Table S2 Search algorithm for Medline, Embase, CENTRAL and Pubmed, Table S3 Studies excluded with reason from full text screening, Table S4 Studies Characteristics, Table S5 Global inconsistency test (mouth opening), Table S6 Global inconsistency test (burning sensation), Table S7 Local inconsistency test: the SIDE approach (node splitting), Figure S1 Loop-specific approach to assess the inconsistency in a network, Figure S2 Forest plot illustrating the mean improvement in mouth opening between lycopene and controls (other interventions), Figure S3 Forest plot illustrating the mean improvement in mouth opening between combination therapy of hyaluronidase and corticosteroids with controls (other interventions), Figure S4 Forest plot illustrating the mean improvement in mouth opening between curcumin and controls (other interventions), Figure S5 Forest plot illustrating the mean improvement in mouth opening between Aloe vera and controls (other interventions), Figure S6 Forest plot illustrating the reduction in burning sensation between Lycopene and controls (other interventions), Figure S7 Forest plot illustrating the reduction in burning sensation between curcumin and controls (other interventions), Figure S8 Funnel plot illustrating the mean improvement in mouth opening between lycopene and controls (other interventions), Figure S9 Funnel plot illustrating the mean improvement in mouth opening between combination therapy of hyaluronidase and corticosteroids with controls (other interventions), Figure S10 Funnel plot illustrating the mean improvement in mouth opening between curcumin and controls (other interventions), Figure S11 Funnel plot illustrating the mean improvement in mouth opening between Aloe vera and controls (other interventions).
