# Supplementary material for: Comparative Efficacy of Interventions for the Management of Oral Submucous Fibrosis: A Systematic Review and Network Meta-Analysis
Source: J Pers Med. 2022 Aug 1;12(8):1272. doi: 10.3390/jpm12081272 (PMC9409832; doi:10.3390/jpm12081272)
Supplement: Supplementary file 1 [file jpm-12-01272-s001.zip › jpm-1808713-supplementary.pdf]

Supporting information

List of tables

Table S1 Search strategy

Table S2 Search algorithm for Medline, Embase, CENTRAL and Pubmed.

Table S3 Studies excluded with reason from full text screening.

Table S4 Studies Characteristics

Table S5 Global inconsistency test (mouth opening)

Table S6 Global inconsistency test (burning sensation)

Table S7 Local inconsistency test: the SIDE approach (node splitting)

List of Figures

Figure S1 Loop-specific approach to assess the inconsistency in a network

Figure S2 Forest plot illustrating the mean improvement in mouth opening between lycopene and controls (other interventions)

Figure S3 Forest plot illustrating the mean improvement in mouth opening between combination therapy of hyaluronidase and corticosteroids with controls (other interventions)

Figure S4 Forest plot illustrating the mean improvement in mouth opening between curcumin and controls (other interventions)

Figure S5 Forest plot illustrating the mean improvement in mouth opening between Aloe vera and controls (other interventions)

Figure S6 Forest plot illustrating the reduction in burning sensation between Lycopene and controls (other interventions)

Figure S7 Forest plot illustrating the reduction in burning sensation between curcumin and controls (other interventions)

Figure S8 Funnel plot illustrating the mean improvement in mouth opening between lycopene and controls (other interventions)

Figure S9 Funnel plot illustrating the mean improvement in mouth opening between combination therapy of hyaluronidase and corticosteroids with controls (other interventions)

Figure S10 Funnel plot illustrating the mean improvement in mouth opening between curcumin and controls (other interventions)

Figure S11 Funnel plot illustrating the mean improvement in mouth opening between Aloe vera and controls (other interventions)

Table S1 Search strategy

**Review question**	In adults with oral submucosal fibrosis, which medical intervention is more effective and safer for its management?
**Population**	Adults receiving medical treatment for oral submucous fibrosis.
**Sub-group**	If heterogenicity is present:<br>1. Drug doses<br>2. Dosing frequency
**Intervention**	Any medical intervention which is used in the management of oral submucous fibrosis, including systemic and topical agents, traditional or complementary medication, as well as other active interventions.
**Comparison**	Placebo, no treatment, or any intervention against those interventions mentioned above (including different doses of the same intervention).
**Outcomes**	***Primary outcome:*** Improvement in mouth opening in patients with oral submucous fibrosis<br><br>***Secondary outcome:*** relief of burning sensation, relief of ddysphagia, adverse events ('probably due to drug'), compliance and development of drug resistance if mentioned by the study
**Study design**	Randomized controlled trials (RCTs)
**Databases**	Medline, Pubmed, Embase, CENTRAL

Table S2 Search algorithm for Medline, Embase, CENTRAL and Pubmed.

**Database**	**Query**		**Items found**
**Medline on Ovid**	1. Oral submucous fibrosis	(oral submucous fibrosis or ("submucous fibrosis" and (oral or mouth)) or (oral submucous fibrosis and ("submucous fibrosis" and (oral or mouth)))).af.	1516
	2. Randomized control trials	(randomized controlled trial or controlled clinical trial or randomized or randomly or random or trial).af.	1949287
	3. Drug therapy	(drug therapy or management or therapy or placebo or treatment).af.	8839234
	4.	#1 AND #2 AND #3	99
**Pubmed**	1. Oral submucous fibrosis	"Oral Submucous Fibrosis"[Mesh] OR "submucous fibrosis" OR "submucous fibroses"	**1548**
	2. Randomized controlled trial	"randomized controlled trial*" OR "randomised controlled trial*" OR "randomized" OR "controlled trial"	**912387**
	3. Drug therapy	"Drug Therapy"[Mesh] OR "treatment" OR "therapy*" OR "management"	**9161947**
	4.	#1 AND #2 AND #3	**68**
**CENTRAL**	1. Oral submucous fibrosis	"Oral Submucous Fibrosis" OR ((submucous NEXT fibrosis) AND (oral or mouth))	139
	2. Randomized controlled trial	"Randomized Controlled trial" OR "controlled clinical trial" OR "randomized"	1320289
	3. Drug therapy	"drug therapy" OR "therapy" OR "management" OR "treatment"	1069702
	4.	#1 AND #2 AND #3	**80**

Table S3 Studies excluded with reason from full text screening.

No	Author, Year	Title	Reason for rejection
1	Li YH et al, 2019	A Comparative Study to Evaluate Efficacy of Curcumin and Aloe Vera Gel along with Oral Physiotherapy in the Management of Oral Submucous Fibrosis: A Randomized Clinical Trial	Ineligible management (not drug therapy
2	Dani et al, 1992	The effectiveness of therapeutic ultrasound in patients with oral submucosal fibrosis	Ineligible management (not drug therapy
3	Lyu FY et al, 2016	Expression of secreted frizzled-related protein-1 in patient with oral submucous fibrosis	Ineligible management (no drug management included)
4	Wu et al, 2010	Therapeutic effect of the combination of traditional Chinese medicine and western medicine on patients with oral submucous fibrosis	Article completely in Chinese language
5	Borle et al, 2009	Extended nasolabial flaps in the management of oral submucous fibrosis	Ineligible management (not drug therapy
6	Cox et al, 2009.	Physiotherapeutic treatment improves oral opening in oral submucous fibrosis	Ineligible management (not drug therapy
7	Khanna et al, 2006	Circulating immune complexes and trace elements (Copper, Iron and Selenium) as markers in oral precancer and cancer: a randomised, controlled clinical trial	Ineligible management (no drug management included)
8	Sun A et al, 2001	Levamisole and/or Chinese medicinal herbs can modulate the serum level of squamous cell carcinoma associated antigen in patients with erosive oral lichen planus.	Ineligible subjects (subjects consist of oral lichen planus patients)

9	Bande C et al, 2016	Immediate versus delayed aggressive physical therapy following buccal fat pad interposition in oral submucous fibrosis-a prospective study in Central India	Ineligible management (not drug therapy)
10	Pardeshi P et al, 2015	Clinical evaluation of nasolabial flap & buccal fat pad graft for surgical treatment of oral submucous fibrosis-a randomized clinical trial on 50 patients in Indian population	Ineligible management (not drug therapy)
11	Ambereen A et al, 2020	Is Coronoidectomy Superior to Coronoidotomy for Improving Maximum Incisal Opening in Patients With Oral Submucous Fibrosis?	Ineligible management (not drug therapy)
12	Ganguly R et al, 2020	Evaluation of efficacy of herbal preparation in the management of oral submucous fibrosis: a study.	No specific management stated
13	Vibha S et al, 2019.	Evaluation of herbal preparation in management of oral submucous fibrosis.	No specific management stated
14	Chloe RH et al, 2011	A randomized controlled trail of lycopene in oral submucous fibrosis.	Outcomes are expressed in the form of percentages
15	Singh M et al, 2010	Efficacy of hydrocortisone acetate/hyaluronidase vs triamcinolone acetonide/hyaluronidase in the treatment of oral submucous fibrosis	No primary outcome available
16	Krishnamoorthy B, 2013	Management of oral submucous fibrosis by two different drug regimens: A comparative study	No values given. Only numberings are provided.
17	Baptist J et al, 2016	Rebamipide to Manage Stomatopyrosis in Oral Submucous Fibrosis	No primary outcome available
18	Daga D et al, 2017	Efficacy of oral colchicine with intralesional hyaluronidase or triamcinolone acetonide in the Grade II oral submucous fibrosis	For primary outcome only mean given and not standard

			deviation and secondary outcome no values were given
19	Rajendran et al, 2006	Pentoxifylline therapy: A new adjunct in the treatment of oral submucous fibrosis	The outcome is expressed in p value only
20	Mehrotra R, 2010	Pentoxifylline Therapy in the Management of Oral Submucous Fibrosis	Only percentage is given in the outcomes
21	Kalkur C et al, 2019	"Introducing Modified Dakkak and Bennett Grading Systemfor Indian Food in Oral Submucous Fibrosis": A Dharwad Study	No primary outcome provided and no baseline value for secondary outcomes
22	Goel S et al, 2015	A comparative study on efficacy of different treatment modalities of oral submucous fibrosis evaluated by clinical staging in population of Southern Rajasthan	The patient groups are divided into stage 1, 2 and 3 and not all patients as a whole
23	Alam S et al, 2010	Efficacy of aloe vera gel as an adjuvant treatment of oral submucous fibrosis	Surgical treatment is one of the treatment options in this study
24	Deepa AD et al, 2010	Comparative study of the efficacy of curcumin and turmeric oil as chemopreventive agents in oral submucous fibrosis: a clinical and histopathological evaluation	No mean and SD provided in the outcomes
25	Singh U et al, 2016	Efficacy and Safety of Intralesional Xantinol Nicotinate in the Treatment of Various Stages of Oral Submucous Fibrosis	No baseline value provided

26	Raizada MK et al, 2017	Omega 3: a novel treatment agent in oral submucous fibrosis: a pilot study	No mean and SD provided in the outcomes
27	Shah A et al, 2018	Comparison of systemic and topical forms of curcumin in oral submucous fibrosis: A clinical and histopathological evaluation in 120 patients	No mean and SD provided in the outcomes
28	Lanjekar AB et al, 2020	Comparison of Efficacy of Topical Curcumin Gel with Triamcinolone-hyaluronidase Gel Individually and in Combination in the Treatment of Oral Submucous Fibrosis	No mean and SD provided in the outcomes
30	Patil et al 2014	Comparison of Alovera and Pentoxyphylline	Outcomes not properly defined
31	Prabhu et al 2015	Pentoxyphylline with conventional therapies including intralesional corticosteroid, hyaluronidase and placentrix injections, along with local heat therapy and mouth stretching exercises	Mouth stretching exercises and heat therapy also included with drug treatment

Table S4: Studies Characteristics

S. No	Author, year, country	Population details		Variables evaluate	Main outcomes
		No/Age	Study groups (intervention and control groups)		
1	Kumar A et al [14], 2007, India	No-58<br>Age-18-70 years	G1 – Lycopene tablets (n=21)<br>G2- Lycopene tablets and intralesional betamethasone (n=19)<br>G3- placebo tablets (n=18)	MO, BS, TP, FB	G1 & G2 (MO & BS) – SSD<br>G3 (MO & BS) –SNSD
2	Karemore T.<br>et al [177]<br>2012, India	No-92<br>Age -17-57 years	Groups - According to staging by Khanna and Andrade<br>G1- Lycopene tablets (n=46)<br>G2- placebo tablets (n=46)	MO, BS, UL	MO & BS (SSD)
3	Sudarshan R et al [18], 2012, India	No – 20 Age – 17-40 years	Groups – According to Pindborg et al<br>G1 – Aloe vera gel (n = 10)<br>G2 – Antioxidant capsules (n=10)	MO, BS, TP, CF	BS – SSD<br>MO, TP, CF - SNSD (with improvement)
4	Bhadage CJ et al [178], 2013, India	No – 40 Age - 18-48(G1)<br>23-45(G2)<br>22-50(G3)	G1 – Isoxsuprine tablets (n=15)<br>G2 – Intralesional dexamethasone + hyaluronidase (n=15)<br>G3 – Placebo tablets	MO, BS	MO-SNSD<br>BS-SSD
5	Selvam et al, 2013 [179], India	No – 45<br>Age – 18-50 years	G1 – lycopene+ intralesional dexamethasone + hyaluronidase (n=15)<br>G2 – antioxidant + intralesional dexamethasone + hyaluronidase (n=15)<br>G3 – Intralesional dexamethasone + hyaluronidase (n=15)	MO, BS	MO – Intragroup SSD<br>Intergroup G1 vs G3; G2 vs G3 SSD<br>BS - SSD
6	Jiang XW et al [6], 2013, China	No – 42 Age – 32.8 years(mean)	G1 – Intralesional triamcinolone (n =14)	MO, BS	No information on statistical

			G2 – Intralesional salvianolic acid B (n = 14)<br>G3 – Intralesional triamcinolone + Salvianolic acid B (n = 14)		difference provided
7	Panigrahi R et al [180], 2014, India	No – 20<br>Age – 22-52 years	G1 – Intralesional triamcinolone + hyaluronidase (n=10)<br>G2 –Intralesional triamcinolone (n=10)	MO, UL, FB	MO, UL, FB - SSD
8	Singh D et al [181], 2014, India	No – 44<br>Age – G1: 29.41 ± 9.11 years G2: 25.59 ± 6.98 years	Group – Classification by Lai DR<br>G1: Lycopene tablets (n=22)<br>G2: Intralesional betamethasone (n=22)	MO, BS	MO, BS - SSD
9	Patil S et al [182], 2014 India	No – 68<br>Age - 30.9 ± 12.8 years (mean)	G1: Spirulina tablets (n=34)<br>G2: Lycopene tablets (n = 34)	MO,BS, UL,P	MO- G2 SSD<br>BS – SNSD
10	Patil S et al [183], 2014 India	No – 42<br>Age - 31.2 ± 12.4 (mean)	G1: spirulina tablets (n=21)<br>G2: Aloe vera gel (n=21)	MO, UL, BS, P	MO, UL – SSD in G1<br>BS, P - SNSD
11	Patil S et al [184], 2014 India	No – 120<br>Age - 31.6±12.7 years (mean)	G1: oxitard capsules (n=60)<br>G2: aloe vera gel (n=60)	MO, BS, TP, P, DS1, DS2	MO, TP, P, DS1, DS2– SSD in G1<br>BS- SNSD
12	Yadav M et al [185], 2014, India	No – 40<br>Age – 32 year (mean)	Group - According to staging by Khanna and Andrade<br>G1: Intralesional dexamethasone + hyaluronidase (n=20)<br>G2: Curcumin + piperine tablets (n=20)	BS, MO, TP	BS -SSD in G2<br>MO-SSD for both end of 1st month<br>TP – SSD for G1 end of 1st month
13	Jiang X et al [186], 2015, China	No – 52<br>Age - 21-35 (G1) 22-37 (G2)	G1: Intralesional triamcinolone (n=26)<br>G2: Intralesional allicin (n=26)	MO, BS	MO- SSD in 16 and 40 weeks for G2

					BS- SSD in 8 and 40 weeks for G2
14	Alora Veedu R et al [120], 2015, India	No – 45<br>Age – 19-60 years	G1: Intralesional Hyaluronidase (n=15)<br>G2: Intralesional dexamethasone (n=15)<br>G3: Intralesional hyaluronidase + intralesional dexamethasone (n=15)	BS, P, MO	BS- SSD but not 3 months after treatment<br>P – SSD in G3<br>MO – SSD but SNSD in month 6
15	Patil S et al [187], 2015 India	No – 120<br>Age - 31.6 ± 12.7 years (mean)	G1: lycopene tablets (n=60)<br>G2: aloe vera gel (n=60)	MO, BS, TP, P, DS1, DS2	MO, TP-SSD in G1<br>BS, P, DS1, DS2 -SNSD
16	Nayak A et al [188], 2015, India	No -72<br>Age - NM	G1: Lycopene tablets (n=24)<br>G2: Lycopene + vitamin E tablets (n=24)<br>G3: Placebo capsules (n=24)	MO, BS, UL	SSD in G1 and G2
17	Arshad O et al [189], 2015, Pakistan	No – 45<br>Age - 36.49 ± 11.82 years	G1: intralesional methylprednisolone (n=15)<br>G2: lycopene capsules (n=15)<br>G3: intralesional methylprednisolone + lycopene capsules (n=15)	MO	MO – SSD for G1 and G3; G2 and G3<br>SNSD for G2 and G3
18	Hazarey VK et al [190], 2015, India	No – 30<br>Age – 18-50 years	G1: curcumin lozenges (n= 15)<br>G2: clobetasol propionate 0.05% ointment (n=15)	MO, BS	MO-SSD for G1<br>BS – SSD for G1 for spicy and normal food
19	Singh N et al [191], 2016, India	No – 40<br>Age – 30-35 years (majority)	G1: aloe vera gel TDS with physiotherapy QID (n=20)<br>G2: antioxidant capsules BD with physiotherapy QID (n = 20)	MO, BS, TP, CP	MO- SSD for both groups but G1 is significantly higher<br>BS – SSD for G1

					TP- SNSD for both but G1 is significantly higher<br>CF- SNSD
20	Kaur H et al [152], 2016, India	No – 30<br>Age – 18-49 years	G1: Lycopene with intralesional dexamethasone and hyaluronidase (n=15)<br>G2: Intralesional dexamethasone and hyaluronidase alone (n=15)	MO, BS	BS – intragroup SSD<br>MO- intragroup SSD for both<br>Intergroup SSD for G1
21	Kopuri RKC et al [192], 2016, India	No – 30<br>Age - >15 years	G1: Lycopene (n=15)<br>G2: Curcumin (n=15)	MO, BS, BL, FB	MO- intragroup SSD<br>Intergroup G1 showed more improvement but not enough to be significant<br>BS- intergroup G2 showed better improvement but do not differ enough to be statistically significant<br>FB- G1 showed better improvement but do not differ enough to be statistically significant<br>BL- G2 showed better reduction but do not differ enough to be statistically significant

22	Pipalia PR et al [193], 2016, India	No – 46<br>Age - 28.2 ± 7.05 (average)	G1: cucurmin + black pepper capsules (n=23)<br>G2: nigella sativa capsules (n=23)	MO, BS, TP, CF	Significance levels not given; the overall response of G1 better than G2
23	Anuradha A et al [161], 2017, India	No -74<br>Age - 32.7 ± 10.3 (mean)	G1: aloe vera juice + aloe vera gel (n=37)<br>G2: intralesional hydrocortisone + hyaluronidase + antioxidant capsules (n=37)	MO, BS, TP, CF	<u>End of 1st month</u><br>BS- significantly higher for G1<br>MO-SNSD<br>CF & TP – significantly higher for G1<br><u>End of 2nd month</u><br>G1 is significantly higher in all parameters<br><u>End of 3rd month</u><br>BS & MO-SNSD<br>CF & TP - SSD
24	Sadaksharam J et al [194], 2017	No – 30<br>Age – 23-54 years	Group - According to staging by Khanna and Andrade<br>G1: Pentoxifylline tablets (n=15)<br>G2: intralesional dexamethasone + hyaluronidase (n=15)	BS, MO	BS- SSD for both<br>MO-SSD for both with G2 with a higher degree of improvement
25	Datarkar A et al [195], 2018, India	No – 64<br>Age – 16-38 years	Group - According to staging by Khanna and Andrade<br>G1: prednisolone mouthwash (n=32)<br>G2: antioxidant capsules (n=32)	MO, BS	MO- G1 significantly higher than G2<br>BS- SSD in G1
26	Patil S et al [196], 2018, India	No – 120<br>Age - 31.6 ± 12.7 years	G1: oxitard capsules (n=60)	MO, BS, TP, P, DS1, DS2	MO, TP – SSD in both

			G2: lycopene capsules (n=60)		P, DS1, DS2-SSD in G1<br>BS-SNSD in both
27	Ara SA et al [197], 2018, India	No-100<br>Age - 25.44 ± 5.43(G1)<br>25.42 ± 7.38(G2)	Group – Classification by Mathur and Jha, Bailoor and Nagesh [209-211]<br>G1: placebo capsules (n=50)<br>G2: curcumin capsules (n=50)	MO, BS, DS1, P, CF, TP	All SSD in G2, SNSD in G1
28	Saran G et al [198], 2018, India	No – 60<br>Age - 26.00 ± 6.43 (G1)<br>27.90 ± 8.66(G2)<br>(mean)	Group - Khanna and Andrade 1995 classification<br>G1: lycopene capsules (n=30)<br>G2: curcumin tablets (n=30)	MO, BS	MO, BS – intragroup SSD<br>Intergroup SNSD<br>G1 shows better MO results than G2
29	Piyush P et al [199], 2018, India	No – 90<br>Age – 17-60 years	G1: cucurmin + piperine tablets (n=30)<br>G2: lycopene capsules (n=30)<br>G3: placebo capsules (n=30)	MO, BS, CF, TP	MO- SSD for G1 vs G3 and G2 vs G3; SNSD for G1 vs G2<br>BS- SSD for G1 vs G3 and G2 vs G3; SNSD for G1 vs G2<br>TP – SNSD<br>CF - SSD for G1 vs G3 and G2 vs G3; SNSD for G1 vs G2
30	Tp B et al [200], 2019, India	No – 60<br>Age – 20-45 years	G1: lycopene (n=20)<br>G2: lycopene + intralesional dexamethasone (n=20)	MO, BS	BS- SSD for G3 by end of 2nd month<br>MO – SSD between groups by 3rd month

			G3: intralesional dexamethasone + hyaluronidase (n=20)		
31	Rai A et al [201], 2019, India	No – 147<br>Age – 16-45 years	G1: antioxidant capsules (n=49)<br>G2: turmeric tablets (n=49)<br>G3: turmeric tablets and turmeric mouthwash (n=49)	MO, BS, TP	SSD for all at 12 weeks<br>BS – SNSD between groups<br>MO – SSD for G2 vs G3<br>TP – SSD for G1 vs G3
32	Rajbhoj AN et al [202], 2021, India	No – 60<br>Age – 15-55 years	Group - Classification by Lai DR<br>G1: curcumin gel (n=30)<br>G2: aloe vera gel (n=30)	BS, MO	BS-SSD intergroup and intragroup with more improvement in G2<br>MO -SNSD intergroup and intragroup with more improvement in G1

BS – Burning Sensation, MO- Mouth Opening, TP – Tongue Protrusion, CF – Cheek Flexibility, P – pain, DS1 – Difficulty in speech, DS2 – Difficulty in swallowing, UL – Ulceration, FB – Fibrous bands, BL – Blanching, SSD- Statistically Significant Difference, SNSD – Statistically Non-Significant Difference, G1 – Group 1, G2- Group 2, G3- Group 3

Table S5: Global inconsistency test (mouth opening)

**Network outcome**	**Chi-square**	**P value for test of global inconsistency**
Efficacy in terms of improving mouth opening	117.04	0.000

Table S6: Global inconsistency test (burning sensation)

**Network outcome**	**Chi-square**	**P value for test of global inconsistency**
Efficacy in terms of relieving burning sensation	15.30	0.0091

Table S7: Local inconsistency test: the SIDE approach (node splitting)

**Trial comparisons**	**Direct comparisons**		**Indirect comparisons**		**Difference**		
	Coef.	Std. Err.	Coef.	Std. Err.	Coef.	Std. Err.	P>z
ALL-CORT *	-2.29	2.30	4.89	724.29	-7.18	724.29	0.99

ALV-ANT	-1.93	1.80	0.75	2.319	-2.68	2.93	0.36
ALV-CUR	0.36	2.32	2.69	1.779	-2.33	2.92	0.43
ALV-HYU+CORT+ANT	0.2	2.80	2.42	2.44	-2.22	3.72	0.55
ALV-LYC	3.3	2.34	1.19	1.40	2.11	2.73	0.44
ALV-OXT	8	2.37	6.201	2.71	1.79	3.60	0.62
ALV-SPL	1.1	2.38	-3.07	2.66	4.17	3.57	0.24
ANT-CORT	-1.61	2.36	1.61	1.97	-3.22	3.07	0.30
ANT-CORT+ANT *	4.27	1.74	5.71	5.95	-1.44	6.20	0.82
ANT-CUR+PEP	-0.69	2.41	1.95	2.36	-2.64	3.37	0.43
CORT-CORT+ANT	2.88	2.35	6.36	3.20	-3.48	3.97	0.38
CORT-CUR	3.27	2.46	2.05	1.75	1.22	3.01	0.69
CORT-HYU*	2.34	2.61	5.44	5.28	-3.10	5.89	0.60
CORT-HYU+CORT *	3.71	1.76	1.70	1.78	2.01	2.50	0.42
CORT-LYC	0.11	1.78	3.90	1.46	-3.79	2.29	0.10
CORT-LYC+CORT	3.75	3.22	3.57	2.04	0.18	3.83	0.96

CUR-PLC	-10.12	1.85	-2.18	1.36	-7.94	2.30	0.00
CUR-LYC	2.15	1.55	-2.11	1.49	4.26	2.15	0.05
CUR+PEP-PLC	-2.43	2.53	-3.10	2.23	0.67	3.35	0.84
CUR+PEP-HYU+CORT	1.88	2.36	2.73	2.12	-0.85	3.17	0.79
CUR+PEP-LYC	0.13	2.58	3.00	1.88	-2.87	3.18	0.37
CUR+PEP-NIG *	-0.25	2.28	-5.63	142.40	5.38	142.42	0.97
HYU-HYU+CORT *	-0.8	2.56	2.31	5.35	-3.11	5.90	0.60
HYU+CORT-PLC *	-3.6	3.26	-5.52	1.56	1.92	3.61	0.60
HYU+CORT-HYU+CORT+ANT	0.90	2.28	-3.11	2.91	4.01	3.69	0.28
HYU+CORT-ISO *	-1.1	3.33	-4.94	7.05	3.84	7.22	0.60
HYU+CORT-LYC	-0.01	1.70	-0.58	1.62	0.48	2.35	0.84
HYU+CORT-LYC+CORT	-0.44	2.33	2.17	2.27	-2.61	3.25	0.42
HYU+CORT-LYC+CORT+HYU *	1.9	2.29	-10.34	237.23	12.24	237.24	0.96

HYU+CORT-PENT *	1.8	2.31	-10.33	597.24	12.13	597.24	0.98
HYU+CORT+ANT-LYC	0.53	2.43	-0.01	2.66	0.54	3.63	0.88
ISO-PLC *	-2.5	3.86	-6.34	6.18	3.84	7.22	0.60
LYC-PLC *	-3.43	1.14	-8.43	1.82	5.00	2.15	0.02
LYC-LYC+CORT *	2.25	1.61	-3.28	3.44	5.54	3.81	0.15
LYC-LYC + VIT E *	0.9	2.44	-0.39	4.81	1.29	5.39	0.81
LYC-OXT	4.7	2.38	6.49	2.70	-1.79	3.60	0.62
LYC-SPL	-4.3	2.35	-0.13	2.690	-4.17	3.57	0.2
LYC+CORT-PLC	-4.54	3.40	-6.58	1.98	2.04	3.93	0.60
LYC+VIT E-PLC *	-5.2	2.39	-6.49	4.88	1.29	5.39	0.81

Abbreviations: HYU- Hyaluronidase; CORT- Corticosteroid; LYC– Lycopene; ALL- Allicin; ANT – Antioxidant; CUR – Curcumin; ALV – Aloe vera; SPL – Spirulina; OXT – Oxitard; VIT E – Vitamin E; PEP – Piperine; NIG – Nigella sativa; PLC – Placebo; ISO – Isoxsuprine; PENT – Pentoxifylline

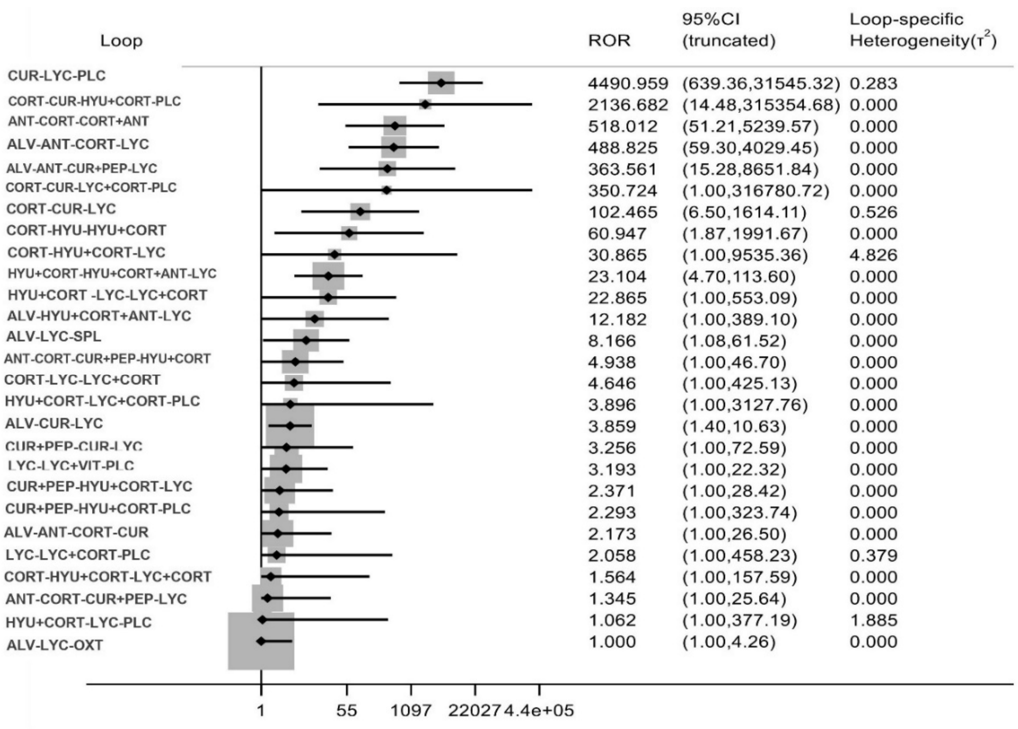

Figure S1 : Loop-specific approach to assess the inconsistency in a network

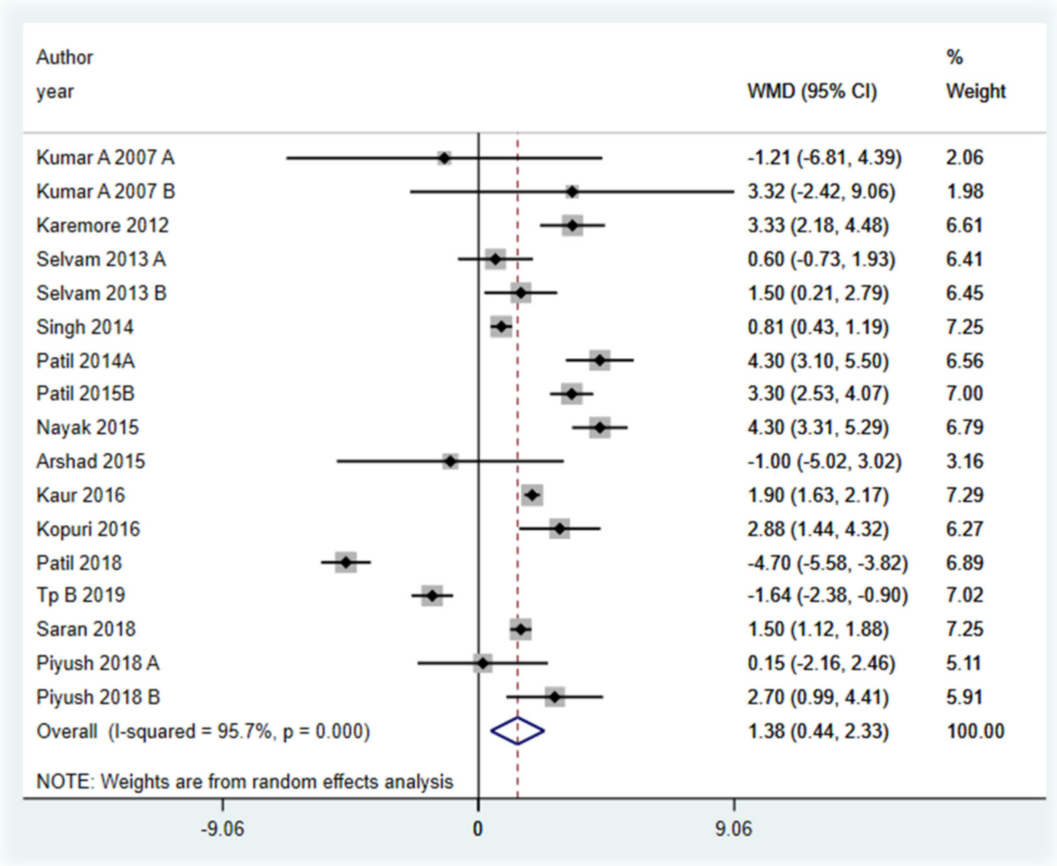

Figure S2: Forest plot illustrating the mean improvement in mouth opening between lycopene and controls (other interventions)

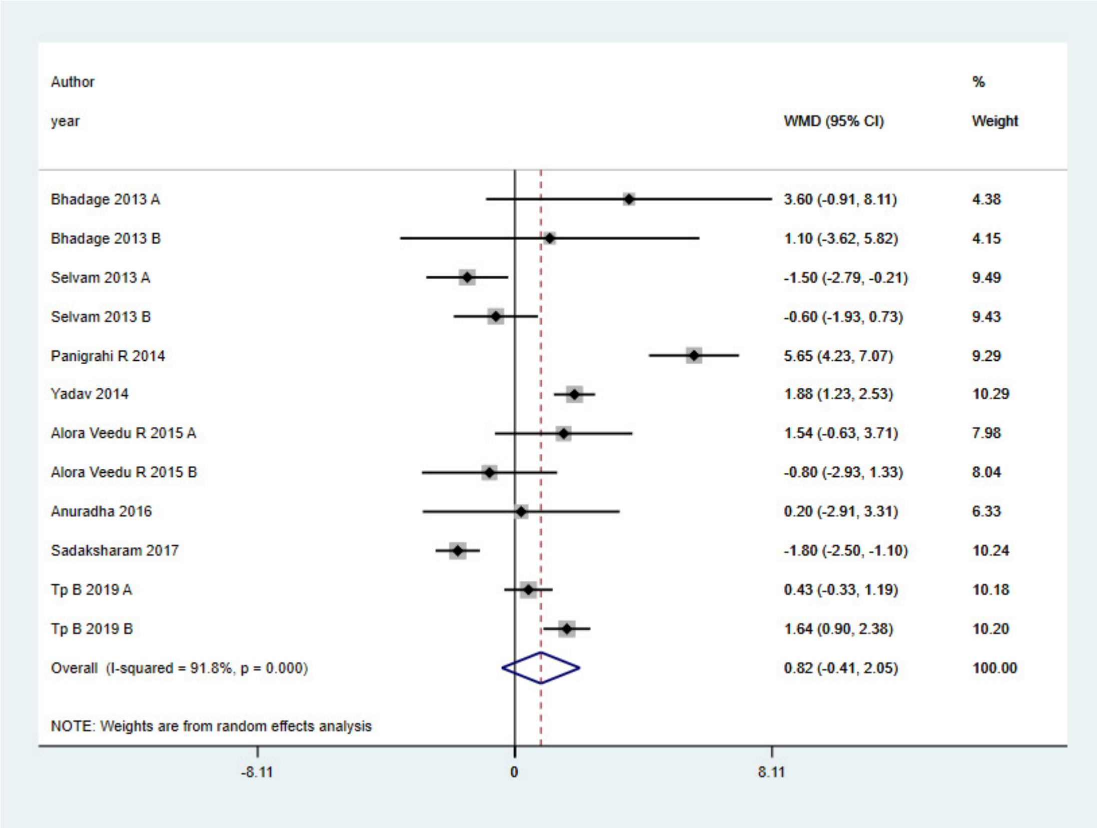

Supplementary Figure S3: Forest plot illustrating the mean improvement in mouth opening between combination therapy of hyaluronidase and corticosteroids with controls (other interventions)

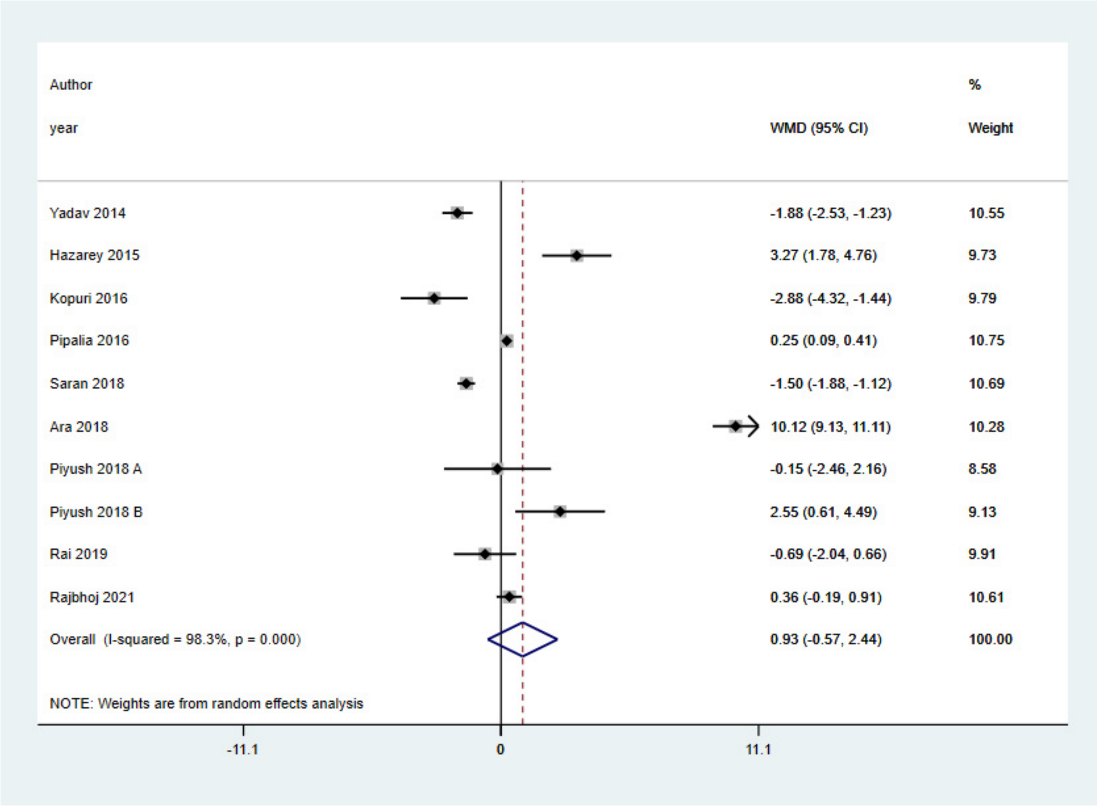

Supplementary Figure S4: Forest plot illustrating the mean improvement in mouth opening between curcumin and controls (other interventions)

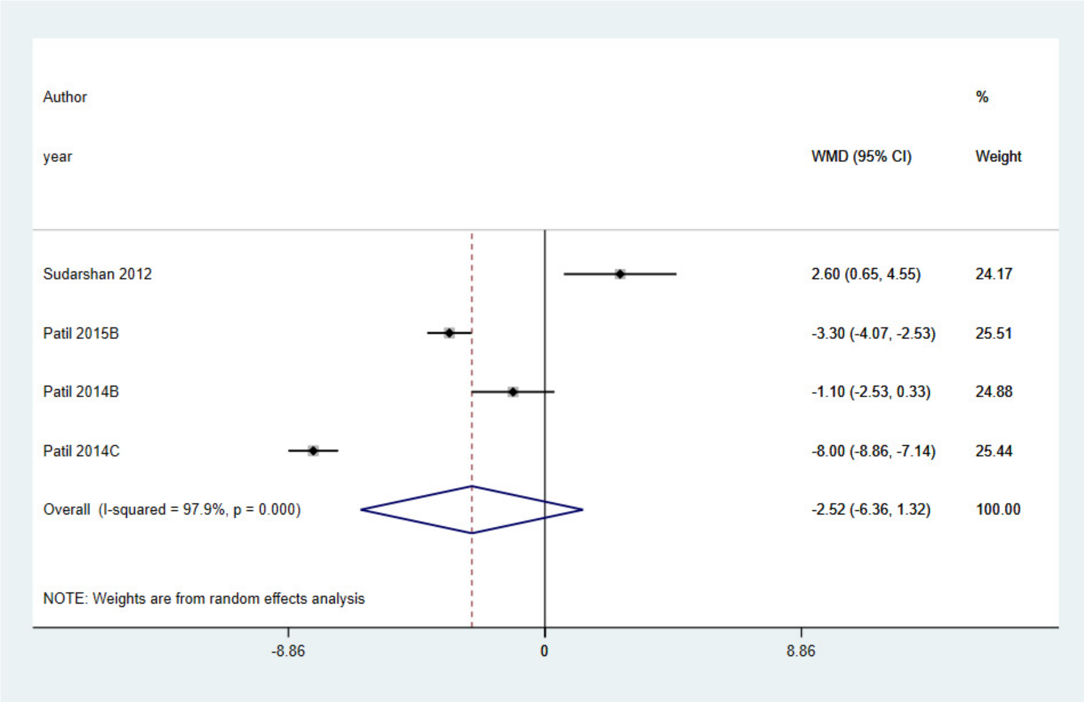

Supplementary Figure S5: Forest plot illustrating the mean improvement in mouth opening between Aloe vera and controls (other interventions)

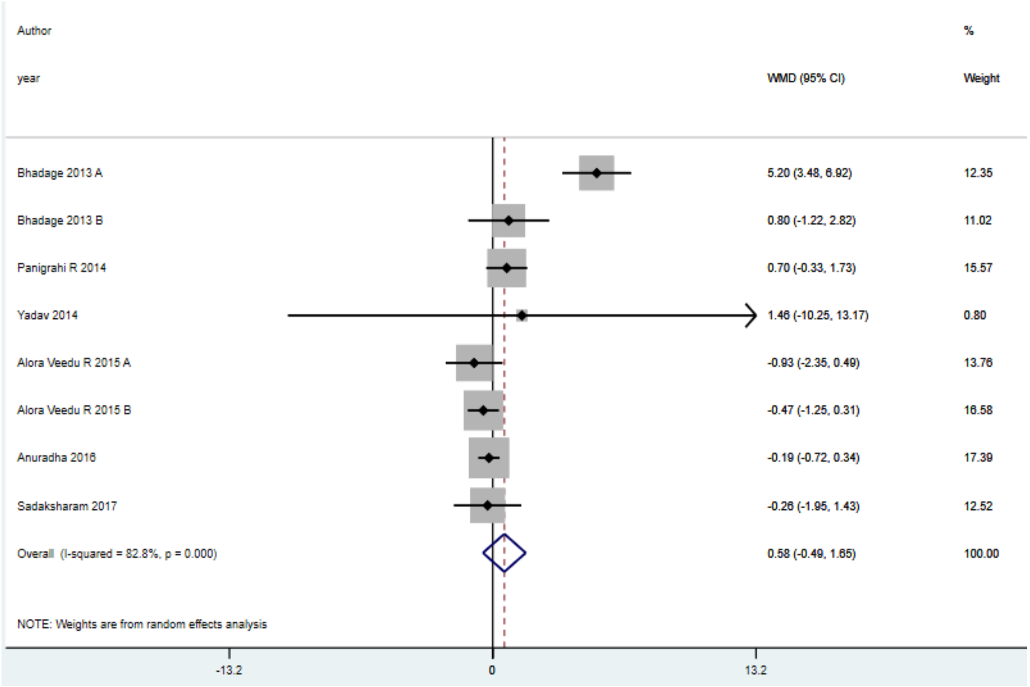

Supplementary Figure S6: Forest plot illustrating the reduction in burning sensation between Lycopene and controls (other interventions)

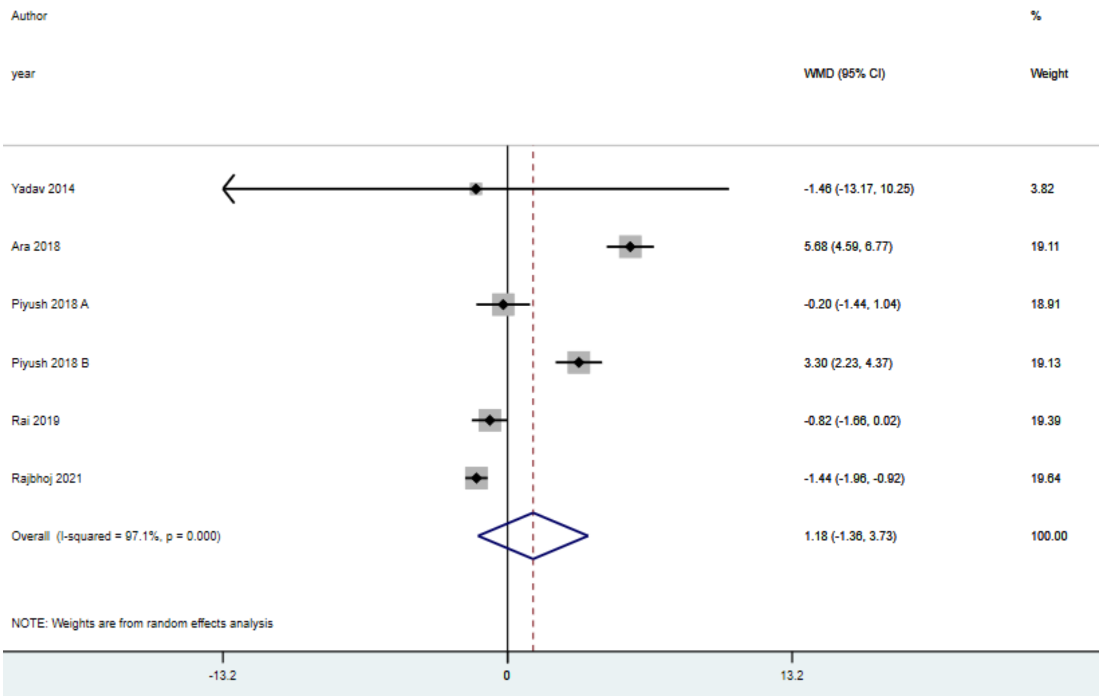

Supplementary Figure S7: Forest plot illustrating the reduction in burning sensation between curcumin and controls (other interventions)

5.8.4.3 Publication bias

Forest plots for all meta-analysis comparisons indicated publication bias.

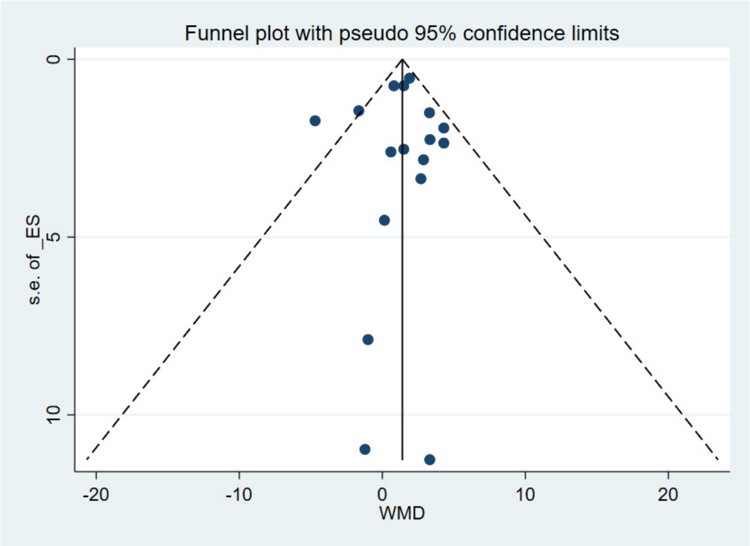

Supplementary Figure S8: Funnel plot illustrating the mean improvement in mouth opening between lycopene and controls (other interventions)

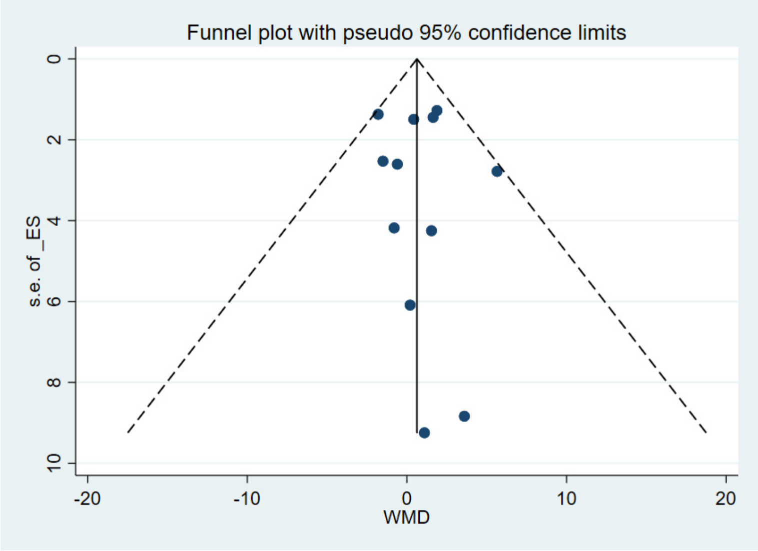

Supplementary Figure S9: Funnel plot illustrating the mean improvement in mouth opening between combination therapy of hyaluronidase and corticosteroids with controls (other interventions)

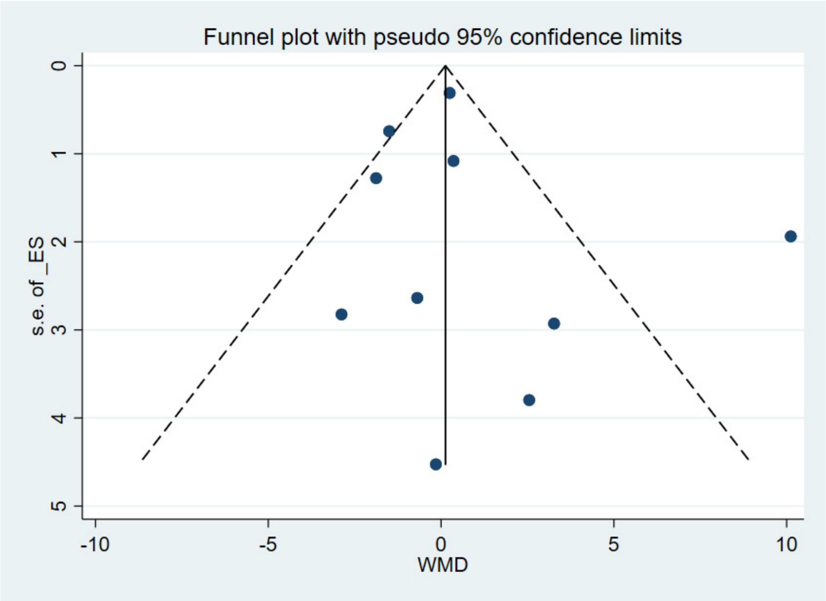

Supplementary Figure S10: Funnel plot illustrating the mean improvement in mouth opening between curcumin and controls (other interventions)

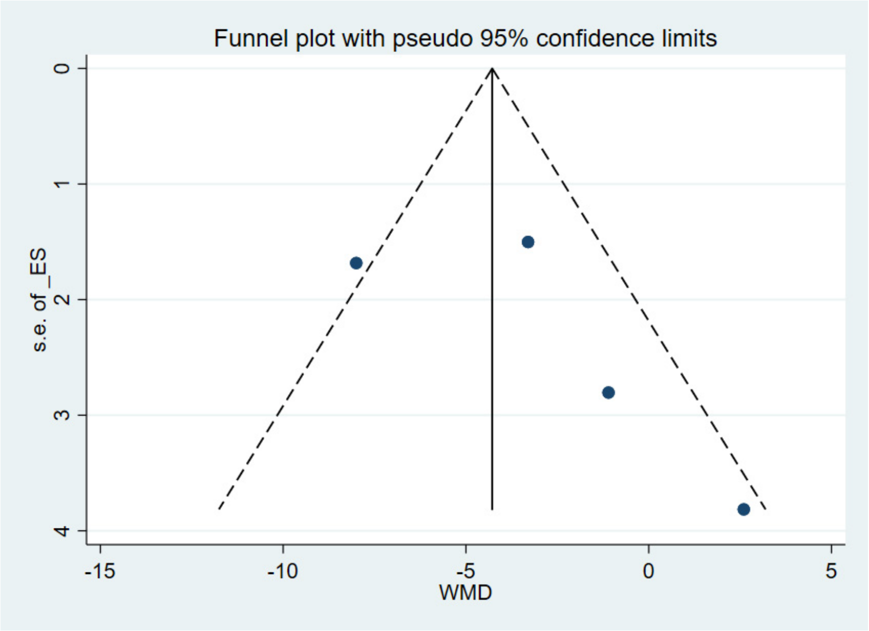

Supplementary Figure S11: Funnel plot illustrating the mean improvement in mouth opening between Aloe vera and controls (other interventions)
